# Current nomenclatural changes in *Cordyceps sensu lato* and its multidisciplinary impacts

**DOI:** 10.1080/21501203.2017.1386242

**Published:** 2017-10-05

**Authors:** Bhushan Shrestha, Gi-Ho Sung, Jae-Mo Sung

**Affiliations:** a Mushtech Cordyceps Institute, Gangwon-do, Korea; b International St. Mary’s Hospital and College of Medicine, Institute for Healthcare and Life Science, Catholic Kwandong University, Incheon, Korea; c Department of Microbiology, College of Medicine, Catholic Kwandong University, Gangneung-si, Korea; d International St. Mary’s Hospital and College of Medicine, Institute for Translational and Clinical Research, Catholic Kwandong University, Incheon, Korea

**Keywords:** *Cordyceps sensu lato*, entomopathogenic fungi, medicinal fungi, nomenclatural changes, one fungus one name, phylogenetic classification

## Abstract

Innumerable name changes have occurred in *Cordyceps* and allied taxa, after the phylogenetic classification of *Cordyceps*, coupled by the application of one fungus one name after the amendment of ICN. Complying with one fungus one name, many generic names have been protected for monophyletic clades in Clavicipitaceae and Ophiocordycipitaceae that have made tremendous transfer of *Cordyceps* spp. to both sexual and asexual genera. Species compositions of the accepted genera *Ophiocordyceps, Tolypocladium, Metarhizium, Perennicordyceps, Polycephalomyces* and *Purpureocillium* are briefly discussed to update the readers with the current placements of *Cordyceps* spp. Some examples of frequent name changes of *Cordyceps* spp. are also mentioned, with reference to use of older scientific names in non-mycological publications.

## Phylogenetic classification of *Cordyceps*

**Figure 1.**
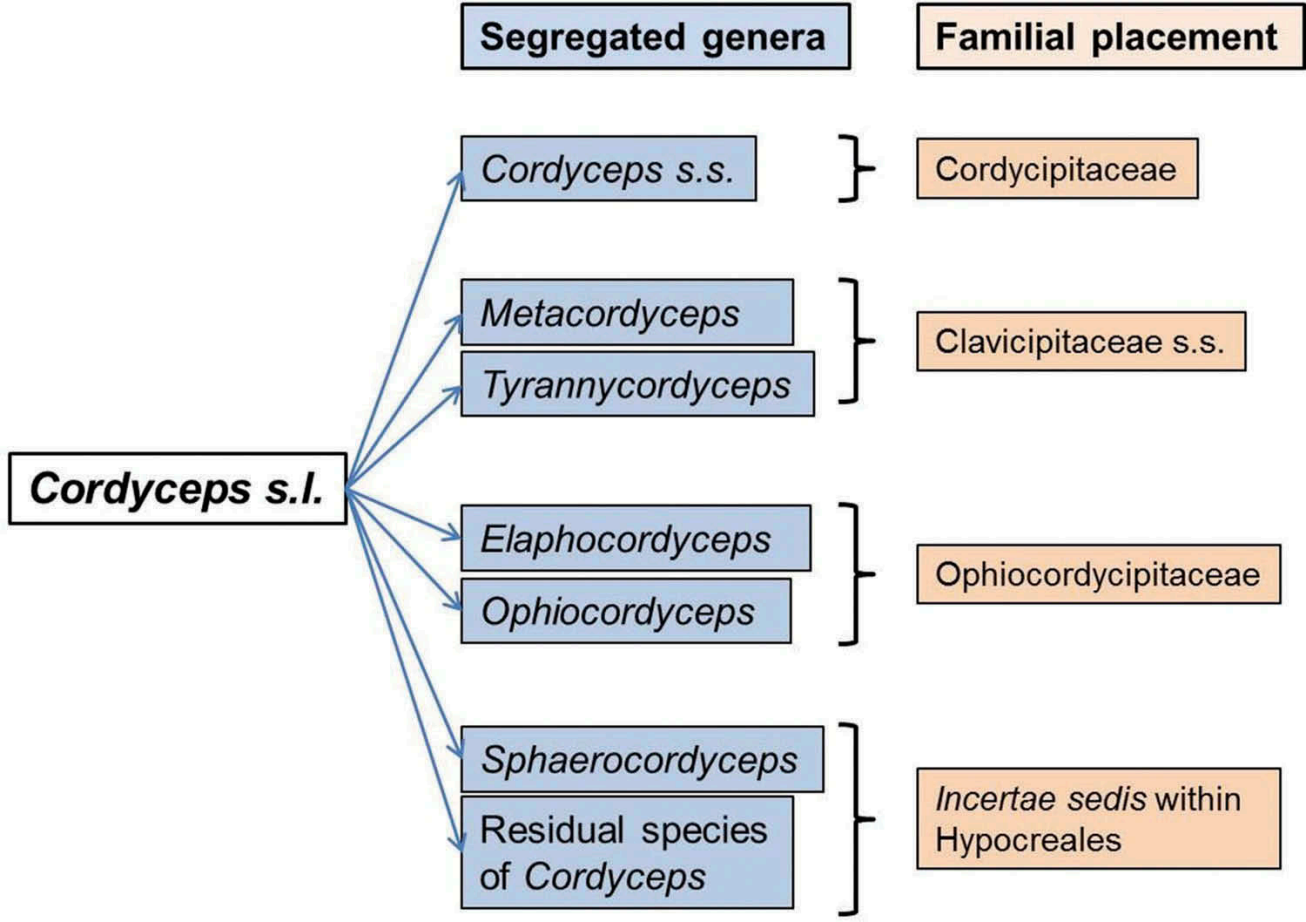
Genera segregated from *Cordyceps s.l.*

The genus *Cordyceps*, established by Fries (1818), was traditionally classified in Clavicipitaceae (Hypocreales, Ascomycota) to accommodate insect and fungal parasites, producing elongated, cylindrical or filamentous stromata with perithecioid type of ascocarp and filamentous, multi-septate ascospores. It is a big hypocrealean genus, comprising more than 400 spp. that parasitise numerous orders of insects, including spiders, in majority (Araújo and Hughes 2016; Shrestha et al. 2016). *Cordyceps* Fr. and allied species are very curious groups of fungi, with long botanical history starting in pre-Linnaean era (Shrestha et al. 2014). Recent molecular phylogenetic studies showed that *Cordyceps sensu lato* is not monophyletic and is intercepted by plant pathogenic genera *Claviceps* Tul., *Balansia* Speg., *Epichloë* (Fr.) Tul. and *C*. Tul. within Clavicipitaceae (Sung et al. 2007). *Cordyceps sensu stricto* was, hence, circumscribed to a clade that consisted of its type species *C. militaris* (L.) Fr., and new genera were proposed for other clades outside *Cordyceps s.s.: Metacordyceps* G.H. Sung et al., *Elaphocordyceps* G.H. Sung et al., *Ophiocordyceps* G.H. Sung et al. and *Tyrannicordyceps* Kepler & Spatafora that together accommodated more than 180 *Cordyceps* spp. (Sung et al. 2007; Kepler et al. 2012b) (Figure 1). Besides transfer of many *Cordyceps* spp. to new genera, many more *Cordyceps* spp. (~ 170 spp.) still remain *incertae sedis* (of uncertain placement) within Hypocreales, because of lack of molecular phylogenetic studies or inconclusive morphological and ecological assessment (Sung et al. 2007).

Among the new genera, *Metacordyceps* shared sister relationship with a clade of plant pathogenic genera (*Claviceps, Balansia, Epichloë*) and were all retained in the family Clavicipitaceae *s.s*. (Sung et al. 2007). *Tyrannicordyceps* is a small genus placed within a clade of *Balansia, Claviceps* and *Epichloë*, to which all five spp. were transferred from *Cordyceps* (Kepler et al. 2012b). All the members of *Tyrannicordyceps* are pathogens of *Claviceps* stromata. *Elaphocordyceps, Ophiocordyceps* and other allied genera formed a separate clade and were placed in a new family Ophiocordycipitaceae that formed a sister clade with Clavicipitaceae *s.s*. (Sung et al. 2007). *Cordyceps s.s*. and other allied genera *Lecanicillium* W. Gams & Zare, *Engyodontium* de Hoog, *Simplicillium* W. Gams & Zare, *Torrubiella* Boud. *s.s*., etc. were placed in a separate family Cordycipitaceae, which formed a sister clade with Hypocreaceae, a family mostly parasitic on other fungi or plants (Sung et al. 2007).

As mentioned above, *Cordyceps s.s*. was circumscribed to a clade in Cordycipitaceae that consists of its type species *C. militaris* (Sung et al. 2007). Chinese caterpillar fungus *Cordyceps sinensis* (Berk.) Sacc., a highly esteemed medicinal herb in traditional Chinese and Ayurvedic medicine that parasitises hepialid larvae in the Tibetan Plateau and the alpine grassland of the Himalayas, was phylogenetically placed in *Ophiocordyceps* (outside *Cordyceps s.s*. clade); hence a new combination *Ophiocordyceps sinensis* (Berk.) G.H. Sung et al. was made with *Cordyceps sinensis* as its synonym (Sung et al. 2007; Shrestha et al. 2010).

## *Cordyceps* and allied genera within hypocreales

*Cordyceps* and allied genera, distributed in three families of Hypocreales (Clavicipitaceae, Ophiocordycipitaceae and Cordycipitaceae), are briefly introduced below (Table 1, Figure 1).

**Table 1.** *Cordyceps* and allied genera distributed in Clavicipitaceae, Cordycipitaceae and Ophiocordycipitaceae.

Family	Sexual (teleomorph) genera	Asexual (anamorph) genera
Clavicipitaceae *s.s*.	*Conoideocrella, Hypocrella, Metacordyceps, Moelleriella, Orbiocrella, Regiocrella, Samuelsia, Tyrannycordyceps*	*Aschersonia, Metarhizium*
Cordycipitaceae	*Ascopolyporus, Cordyceps* s.s., *Hyperdermium, Torrubiella* s.s.	*Akanthomyces, Beauveria, Engyodontium, Gibellula, Isaria, Lecanicillium, Microhilum, Parengyodontium, Simplicillium*
Ophiocordycipitaceae	*Elaphocordyceps, Ophiocordyceps, Podocrella*	*Drechmeria, Harposporium, Hirsutella, Hymenostilbe, Paraisaria, Polycephalomyces, Purpureocillium, Syngliocladium, Tolypocladium*

### Clavicipitaceae

Besides *Metacordyceps* and *Tyrannicordyceps*, six other sexual genera *Conoideocrella* D. Johnson et al., *Hypocrella* Sacc., *Moelleriella* Bres., *Orbiocrella* D. Johnson et al., *Regiocrella* P. Chaverri & K.T. Hodge and *Samuelsia* P. Chaverri & K.T. Hodge are placed in this family (Sung et al. 2007; Chaverri et al. 2008; Johnson et al. 2009) (Table 1). All of them are parasitic on scale insects or white flies. Similarly, asexual genera placed in this family are *Aschersonia* Mont. and *Metarhizium* Sorokīn (Sung et al. 2007) (Table 1). Among teleomorphic genera, *Conoideocrella* and *Orbiocrella* were formerly classified in *Torrubiella s.l*. (Johnson et al. 2009). Other four genera *Hypocrella, Moelleriella, Regiocrella* and *Samuelsia* are linked to *Aschersonia* or *Aschersonia*-like anamorphs (Chaverri et al. 2005a, 2008).

### Cordycipitaceae

Besides *Cordyceps s.s*., three other sexual genera are placed in this family, *Ascopolyporus* Möller, *Hyperdermium* J. White et al. and *Torrubiella s.s*. (Sung et al. 2007; Johnson et al. 2009) (Table 1). They are parasites of scale insects or spiders. Similarly, nine asexual genera placed in this family are *Akanthomyces* Lebert, *Beauveria* Vuill., *Engyodontium, Gibellula* Cavara, *Isaria* Pers., *Lecanicillium, Microhilum* H.Y. Yip & A.C. Rath, *Parengyodontium* C.-C. Tsang et al. and *Simplicillium* (Sung et al. 2007; Johnson et al. 2009; Vega et al. 2012; Tsang et al. 2016) (Table 1). Among them, *Akanthomyces, Beauveria, Isaria, Lecanicillium* and *Microhilum* are linked to *Cordyceps*, and *Gibellula* is linked to *Torrubiella s.s*.

### Ophiocordycipitaceae

*Elaphocordyceps, Ophiocordyceps* and *Podocrella* Seaver are sexual genera placed in Ophiocordycipitaceae (Sung et al. 2007; Kirk et al. 2013) (Table 1). Anamorphic genera placed in this family are *Drechmeria* W. Gams & H.B. Jansson, *Harposporium* Lohde, *Hirsutella* Pat., *Hymenostilbe* Petch, *Paraisaria* Samson & B.L. Brady, *Polycephalomyces* Kobayasi, *Purpureocillium* Luangsa-ard et al., *Syngliocladium* Petch and *Tolypocladium* W. Gams (Sung et al. 2007; Luangsa-Ard et al. 2011; Quandt et al. 2014) (Table 1). Among them, *Hirsutella, Hymenostilbe, Paraisaria* and *Syngliocladium* are linked to *Ophiocordyceps* (Quandt et al. 2014). Among *Elaphocordyceps* spp., only *E. subsessilis* (Petch) G.H. Sung et al. is known to have *Tolypocladium* anamorph (Quandt et al. 2014). *Podocrella* is linked to *Harposporium*, which is mainly known from nematodes (Chaverri et al. 2005b).

## Current placement of *Cordyceps* spp. following amendment of article 59 of ICN (one fungus one name)

**Figure 2.**
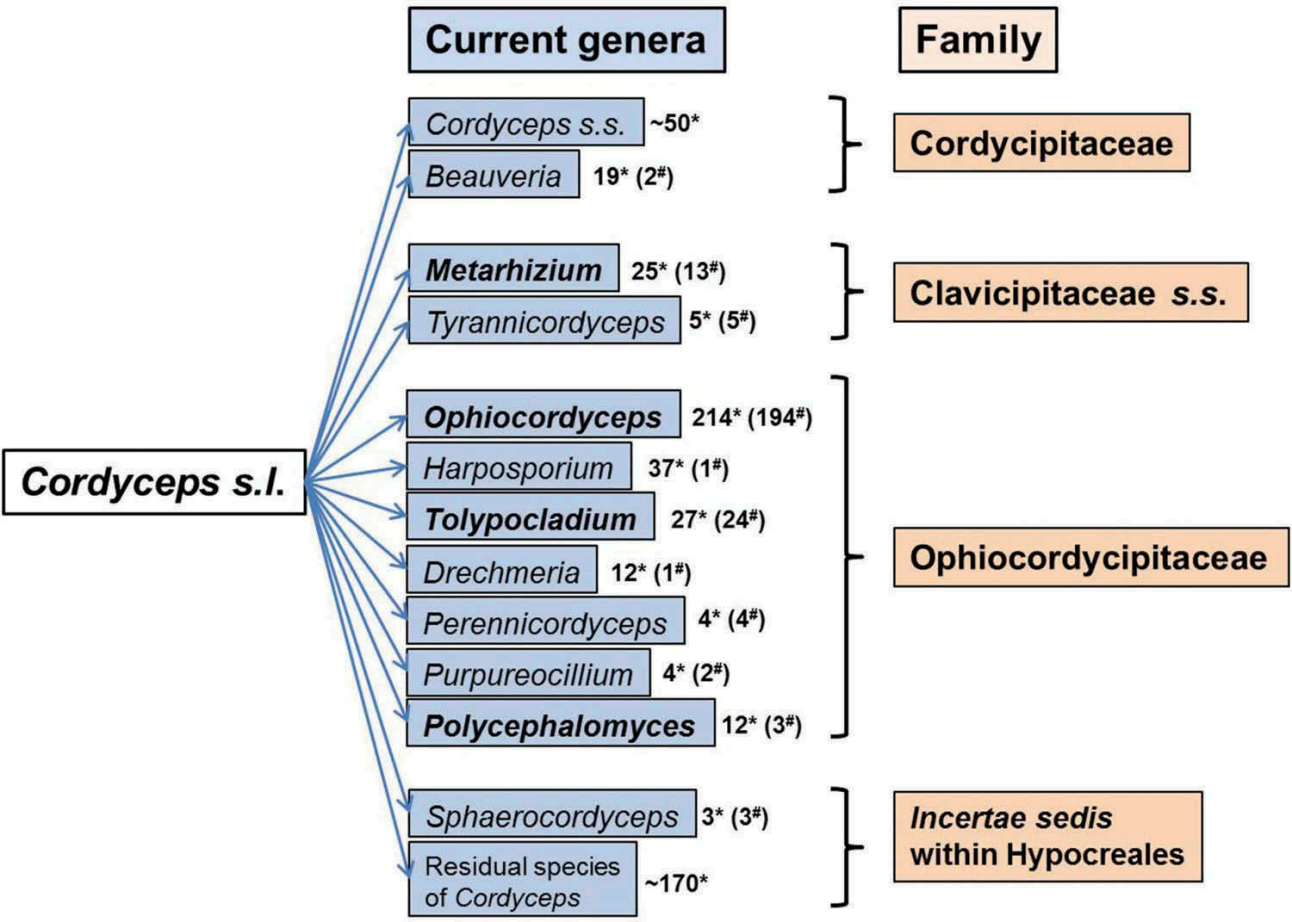
*Cordyceps* spp. transferred to other genera following one fungus one name. * = total no. of spp. accommodated in a genus, ^#^ = no. of spp. transferred from *Cordyceps.*

It is clear from above that the genus *Cordyceps* was split into several phylogenetic genera and many name changes occurred after transfer of *Cordyceps* spp. to new genera (Sung et al. 2007; Kepler et al. 2012b). However, the name changes did not stop there. In April 2011, Amsterdam Declaration decided on *one fungus one name* that eventually amended Art. 59 of the Botanical Code (renamed as International Code of nomenclature for algae, fungi, and plants (ICN) by the 18^th^ International Botanical Congress held in Melbourne in July 2011, also known as Melbourne Code) to eliminate the dual naming of fungi typified by their sexual and asexual states, effective on 1 January 2013 (Hawksworth et al. 2011; McNeill et al. 2012). The newly amended ICN has significant implications on plant pathogenic fungi (Wingfield et al. 2012; Zhang et al. 2013), medically important fungi (De Hoog et al. 2015) as well as hypocrealean entomopathogenic fungi (Kepler et al. 2013, 2014; Quandt et al. 2014; Spatafora et al. 2015; Humber 2016). Following one fungus one name, only a single generic name regardless of its state will be protected or accepted for a monophyletic clade against all other generic names available in that clade based on nomenclatural priority in principle, so that a single scientific name can be given to a single species. Recently, single generic names have been protected for monophyletic clades of invertebrate pathogens in Ophiocordycipitaceae and Clavicipitaceae. The protected names that accommodate former *Cordyceps* spp. are briefly discussed below (Figure 2).

### Cordycipitaceae

*Cordyceps s.s*. currently comprises around 50 spp. (Figure 2). Recently, two new spp. with cordyceps-like sexual states were named as *Beauveria* spp. (*Beauveria gryllotalpidicola* Luangsa-ard et al. and *B. loeiensis* Luangsa-ard et al.) based on their phylogenetic placement in *Beauveria* clade within *Cordyceps* (Ariyawansa et al. 2015) (Figure 2).

### Ophiocordycipitaceae

Complying with one fungus one name, many sexually and asexually typified generic names are protected for monophyletic clades in Ophiocordycipitaceae. They are *Drechmeria, Harposporium, Ophiocordyceps, Perennicordyceps* Matočec & Kušan, *Polycephalomyces, Purpureocillium* and *Tolypocladium* (Kepler et al. 2013; Matočec et al. 2014; Quandt et al. 2014; Spatafora et al. 2015). All the protected genera in Ophiocordycipitaceae include *Cordyceps* spp. Species compositions of the protected genera are briefly discussed below.

### Ophiocordyceps

**Figure 3.**
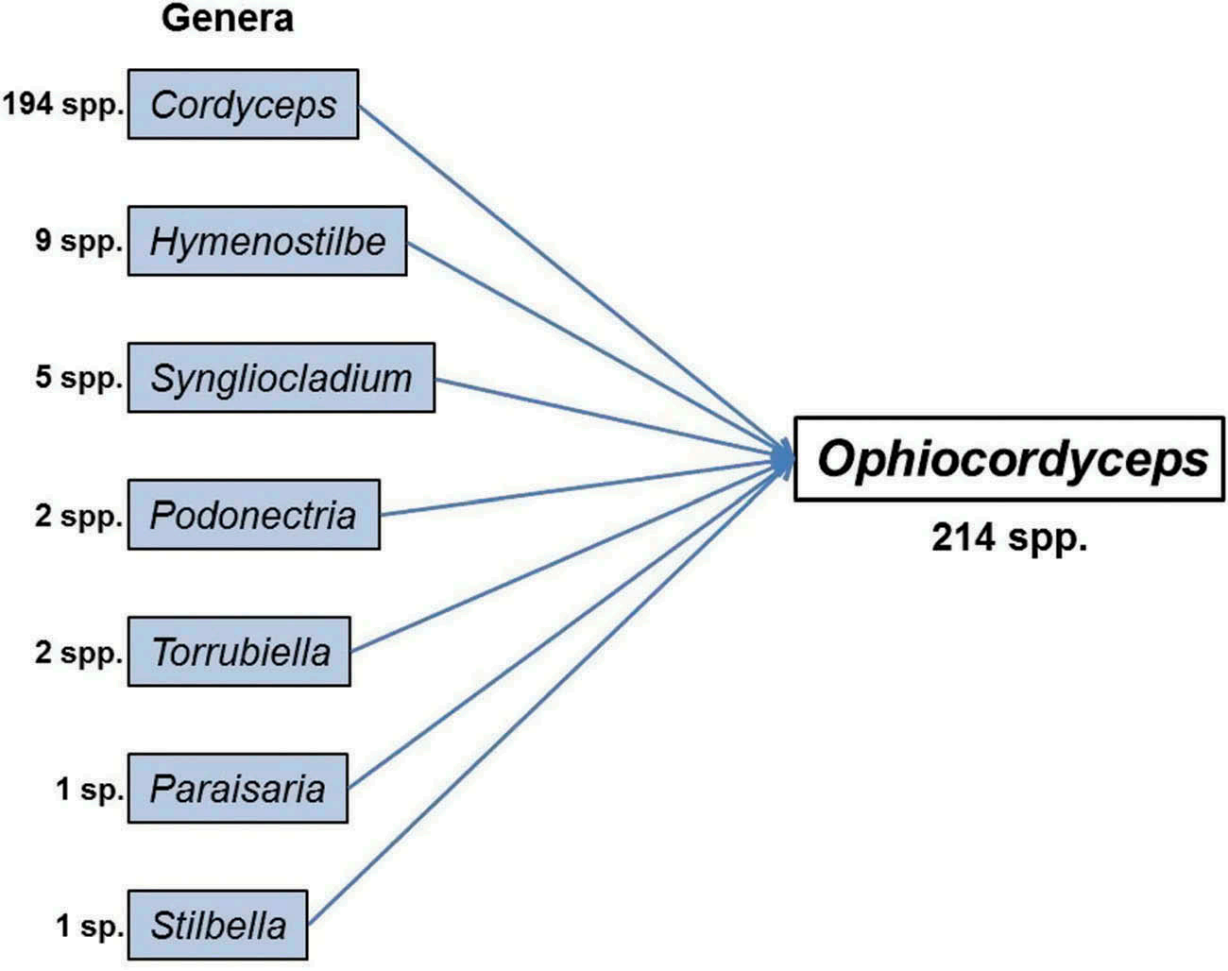
Species composition of *Ophiocordyceps.*

It is the largest genus in Ophiocordycipitaceae with 214 spp. Among them, 194 spp. are transferred from *Cordyceps* or are typified by cordyceps-like sexual states (Figure 2). Besides *Cordyceps* or cordyceps-like spp., *Ophiocordyceps* includes other spp. transferred from sexual and asexual genera, based on their phylogenetic placement (Figure 3). Among sexual spp., two spp. from each *Podonectria* Petch and *Torrubiella* were transferred to *Ophiocordyceps* (Spatafora et al. 2015) (Figure 3). Similarly, many other asexually typified spp. were transferred to *Ophiocordyceps* such as nine *Hymenostilbe* spp., five *Syngliocladium* spp. and one sp. from each *Paraisaria* and *Stilbella* Lindau (Spatafora et al. 2015) (Figure 3). *Ophiocordyceps* thus consists of both sexually and asexually typified spp. For such genera as *Ophiocordyceps, Metarhizium, Tolypocladium*, alternative or suppressed generic names retain the role of morphological descriptors as suggested by Gams (2016).

### Tolypocladium

**Figure 4.**
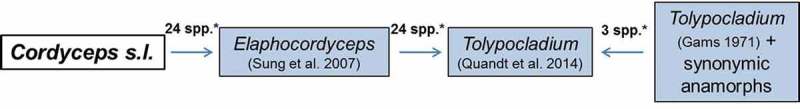
Species composition of *Tolypocladium*. * indicates the no. of spp. transferred from one genus to another.

As mentioned above, *Elaphocordyceps* was erected to accommodate 24 *Cordyceps* spp., mostly growing on *Elaphomyces* fungi, and few on cicada nymphs and coleopteran larva (Sung et al. 2007). One of them, *Elaphocordyceps subsessilis*, is linked to *Tolypocladium, T. inflatum* W. Gams. *Tolypocladium* was originally established by Gams (1971) to encompass soil-borne asexual fungi and currently consists of 27 spp. (Figure 2). In order to comply with one fungus one name, *Tolypocladium* was protected against *Elaphocordyceps* and *Chaunopycnis* W. Gams, another asexually typified genus in the same clade (Quandt et al. 2014) (Figure 4). All 24 *Elaphocordyceps* spp. (including *E. subsessilis*) and 3 *Chaunopycnis* spp. were transferred to *Tolypocladium* (Quandt et al. 2014) (Figures 2 and 4).

### Perennicordyceps

**Figure 5.**
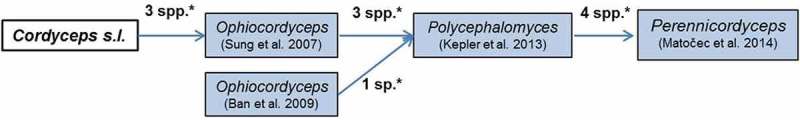
Species composition of *Perennicordyceps*. * indicates the no. of spp. transferred from one genus to another.

Matočec and Kušan established a new genus *Perennicordyceps* to encompass four spp. in *Polycephalomyces* (Matočec et al. 2014) (Figures 2 and 5). All four spp. in *Perennicordyceps* were previously classified in either *Cordyceps* (*C. cuboidea* Kobayasi & Shimizu, *C. prolifica* Kobayasi and *C. ryogamiensis* Kobayasi & Shimizu) or *Ophiocordyceps* (*O. paracuboidea* S. Ban et al.) (Figure 5). All spp. were first placed in *Ophiocordyceps* (Sung et al. 2007; Ban et al. 2009) and then transferred to *Polycephalomyces* (Kepler et al. 2013) prior to transfer to *Perennicordyceps* (Matočec et al. 2014) (Figure 5).

### Purpureocillium

*Purpureocillium* was recently erected to delimit a *Paecilomyces* sp., *P. lilacinus* (Thom) Samson and closely allied taxa (Luangsa-Ard et al. 2011). Currently, there are four spp. in *Purpureocillium* (Figure 2). Among them, *C. ryogamimontana* Kobayasi & Shimizu (current name *Purpureocillium takamizusanense* (Kobayasi) S. Ban et al.) and *C. cylindrica* Petch (current name *Purpureocillium atypicolum* (Petch) Spatafora et al.) were recently transferred from *Cordyceps* based on their phylogenetic placement in *Purpureocillium* (Luangsa-Ard et al. 2011; Ban et al. 2015; Spatafora et al. 2015).

### Polycephalomyces

**Figure 6.**
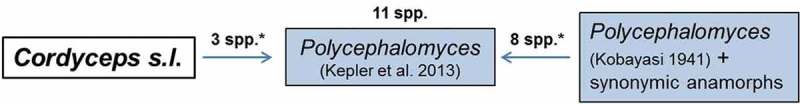
Species composition of *Polycephalomyces*. * indicates the no. of spp. transferred from one genus to another.

It is an asexually typified genus proposed by Kobayasi (1941). It was recently amended by Kepler et al. (2013) and more recently by Matočec et al. (2014) in more strict sense. Currently, there are 11 spp. in this genus, of which three spp. were transferred from *Cordyceps, C. kanzashiana* Kobayasi & Shimizu (current name *Polycephalomyces kanzashianus* (Kobayasi & Shimizu) Kepler & Spatafora), *C. nipponica* Kobayasi (current name *P. nipponicus* (Kobayasi) Kepler & Spatafora) and *C. ramosopulvinata* Kobayasi & Shimizu (current name *P. ramosopulvinatus* (Kobayasi & Shimizu) Kepler & Spatafora) (Kepler et al. 2013) (Figures 2 and 6).

### Drechmeria and harposporium

*Drechmeria* was originally established by Gams and Jansson (1985) to accommodate asexual endoparasitic nematophagous fungi. It currently consists of 12 spp., including one *Cordyceps* sp., *C. gunnii* (Berk.) Berk. (current name *Drechmeria gunnii* (Berk.) Spatafora et al.) (Spatafora et al. 2015) (Figure 2). *Harposporium* is also an asexually typified genus, originally described by Lohde (1874) for nematophagous fungi. It currently consists of 37 spp., with one sp. transferred from *Cordyceps, C. peltata* Wakef. (current name *Harposporium peltatum* (Wakef.) Spatafora & Kepler) based on its phylogenetic placement (Spatafora et al. 2015) (Figure 2).

### Clavicipitaceae

**Figure 7.**
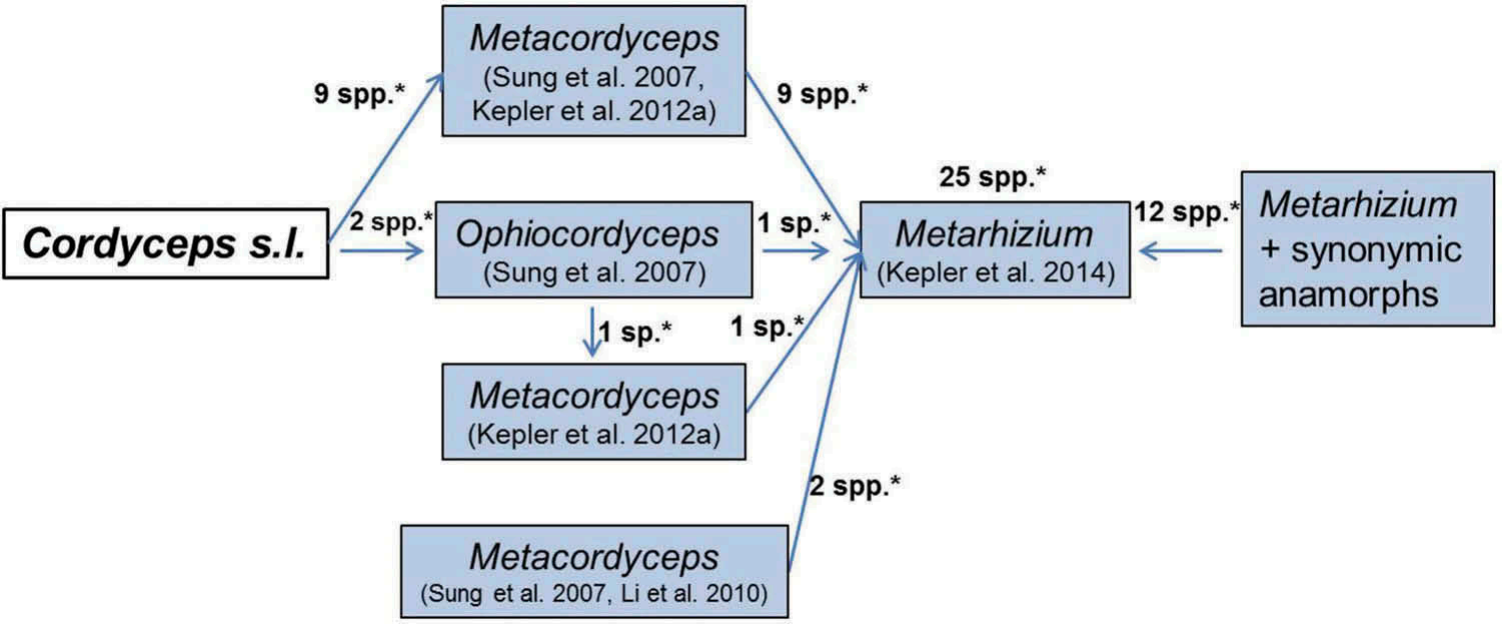
Species composition of *Metarhizium*. * indicates the no. of spp. transferred from one genus to another.

*Metarhizium* is a protected generic name in Clavicipitaceae that comprises spp. previously classified in *Cordyceps. Metarhizium* currently consists of 25 spp. Among them, 13 spp. were transferred from *Cordyceps* or *Metacordyceps* to *Metarhizium*, complying with one fungus one name (Figures 2 and 7). As shown in Figure 7, among 11 spp. transferred from *Cordyceps*, nine spp. were first transferred from *Cordyceps* to *Metacordyceps* prior to transfer to *Metarhizium* (Sung et al. 2007; Kepler et al. 2012a, 2014). Another sp. was first transferred from *Cordyceps* to *Ophiocordyceps* and then finally to *Metarhizium* (Sung et al. 2007; Kepler et al. 2014). The remaining sp. was first transferred from *Cordyceps* to *Ophiocordyceps* and then to *Metacordyceps* prior to transfer to *Metarhizium* (Sung et al. 2007; Kepler et al. 2012a, 2014). Two more *Metacordyceps* spp. were transferred to *Metarhizium*, following one fungus one name (Kepler et al. 2014) (Figure 7).

### Incertae sedis within hypocreales

Besides nearly 170 *Cordyceps* spp. *incertae sedis* within Hypocreales, *Sphaerocordyceps* Kobayasi also belongs to this group (Kobayasi 1981; Sung et al. 2007) (Figure 2). *Sphaerocordyceps* comprises three spp., all transferred from *Cordyceps, C. helopis* Quél. (current name *S. helopis* (Quél.) Kobayasi), *C. palustris* Berk. & Broome (current name *S. palustris* (Berk. & Broome) Kobayasi) and *C. ussuriensis* Koval (current name *S. ussuriensis* (Koval) Kobayasi).

## Impact of one fungus one name on nomenclature of *Cordyceps* spp.

The use of entomopathogenic fungi is getting wider due to their economic and environmental importance. Several species of *Cordyceps* are highly regarded as medicinal herbs in oriental medicine in Asia and have been successfully cultivated for commercial application. Other entomopathogenic fungi, asexual spp. in particular, have been successfully used for biological control of insects and pests. Professionals such as biochemists, pharmacologists, alternative (traditional) medicine practitioners, drug researchers, biocontrol researchers, insect pathologists, forest pathologists and entomologists are widely involved in the research and use of entomopathogenic fungi, besides mycologists.

To cope with name changes of fungal spp., different authors have suggested for the smooth application of one fungus one name for the benefit of their user groups, such as plant pathogenic fungi and medically important fungi (Wingfield et al. 2012; Zhang et al. 2013; De Hoog et al. 2015). De Hoog et al. (2015) have cautioned that nomenclatural changes of medically important fungi may take decades to gain wide acceptance and have suggested some delay in following name changes. With respect to hypocrealean invertebrate-parasitic fungi, Kepler et al. (2013, 2014), Quandt et al. (2014) and Spatafora et al. (2015) have vastly contributed to the application of one fungus one name.

**Figure 8.**
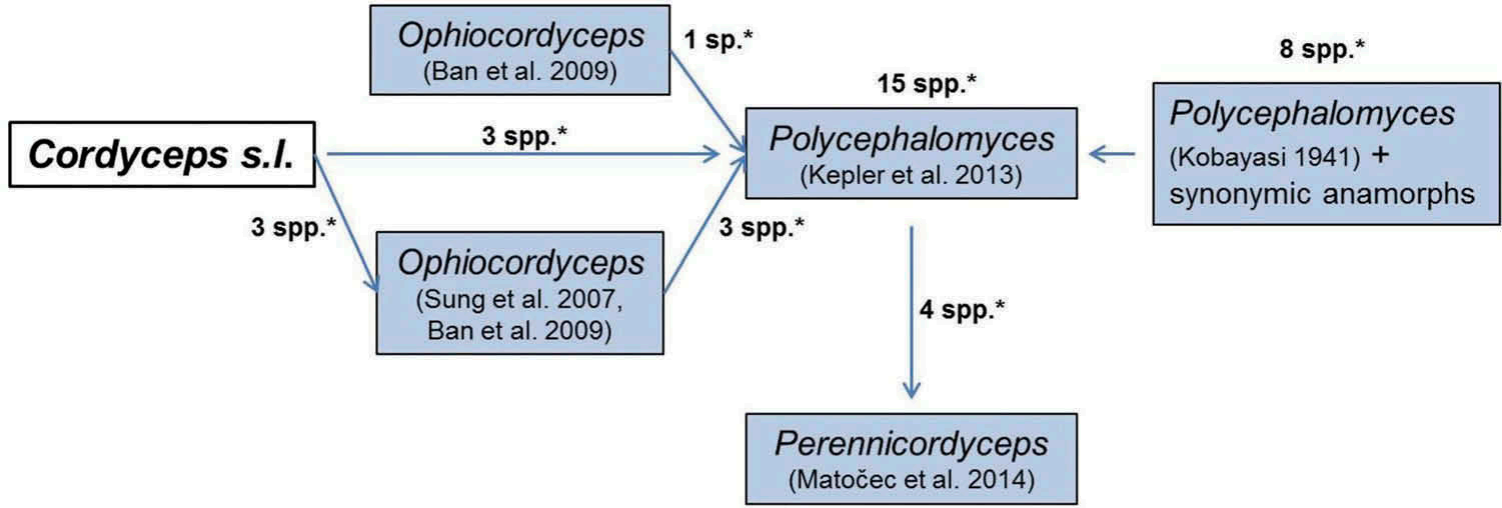
Multiple transfers of *Cordyceps* spp. from one genus to another. * indicates the no. of spp. transferred from one genus to another.

The name changes have been two-fold for hypocrealean invertebrate-parasitic fungi in recent years that have caused multiple name changes within a short duration in some cases: the first one based on the phylogenetic arrangement of *Cordyceps* spp. and the second one based on the application of one fungus one name. The phylogenetic arrangement of *Cordyceps* spp. has been discussed above. The name changes following one fungus one name are more diverse. They are briefly discussed here with reference to multiple name changes from *Cordyceps* to *Perennicordyceps*, for instance (Figure 8). *Cordyceps cuboidea* and *C. ryogamiensis* were transferred to *Ophiocordyceps* following the phylogenetic split of genus *Cordyceps* and were renamed as *Ophiocordyceps cuboidea* and *O. ryogamiensis*, respectively (Sung et al. 2007). After the application of one fungus one name, they were again named as *Polycephalomyces cuboideus* and *Po. ryogamiensis* as *Polycephalomyces* was protected for a clade where *O. cuboidea* and *O. ryogamiensis* were placed (Kepler et al. 2013). However, the clade that included *Polycephalomyces cuboideus* and *Po. ryogamiensis* was again delineated as a separate genus *Perennicordyceps* and consequently the spp. were renamed as *Perennicordyceps cuboidea* and *Pe. Ryogamiensis,* respectively (Matočec et al. 2014). De Hoog et al. (2015) recently opined that when clade system is used for naming, there is no delimitation criterion and that when a genus becomes nearly congruent to species, then it becomes a redundant rank.

*Perennicordyceps cuboidea* (Kobayasi & Shimizu) Matočec et al. (2014)

Basionym: *Cordyceps cuboidea* Kobayasi & Shimizu (1980)

Synonyms: *Ophiocordyceps cuboidea* (Kobayasi & Shimizu) S. Ban et al. (2009).

*Polycephalomyces cuboideus* (Kobayasi & Shimizu) Kepler et al. (2013)

*Perennicordyceps ryogamiensis* (Kobayasi & Shimizu) Matočec et al. (2014)

Basionym: *Cordyceps ryogamiensis* Kobayasi & Shimizu (1983)

Synonyms: *Ophiocordyceps ryogamiensis* (Kobayasi & Shimizu) G.H. Sung et al. (2007)

*Polycephalomyces ryogamiensis* (Kobayasi & Shimizu) Kepler et al. (2013)

Minnis (2015) has rightly pointed out that due to frequent name changes, the users of fungal names get frustrated. Few examples are shown here where older names of *Cordyceps* spp. are being used in non-mycological publications despite nomenclatural changes. *Cordyceps sinensis* was established by Saccardo (1883) during the taxonomic revision of *Cordyceps* that was recently transferred to *Ophiocordyceps* resulting in *O. sinensis* as its currently accepted name, after the phylogenetic classification of *Cordyceps* (Sung et al. 2007) and is now widely accepted by the mycological community. However, the older name *C. sinensis* is still in frequent use in non-mycological publications (Yan et al. 2014; Yan and Wu 2014; Yu et al. 2016), despite its recent nomenclatural change. Similarly, *C. ophioglossoides* (Sun et al. 2014) and *C. sobolifera* (Yang and Zhang 2016) are used in publications, in spite of their recent nomenclatural changes. Though non-mycologists know the name changes, they may ignore in publications. Editors or reviewers of non-mycological journals may also simply not be aware of current nomenclatural changes of fungal species or may not put much emphasis on names changes. It is also true that non-mycologists may not be aware of worldwide online databases of fungi such as MycoBank, IndexFungorum and Fungal Names to be update with the fungal name changes. The more rapidly the name changes take place, other professionals will simply feel safe by using older names.
